# Supplementary figures and images for: Cell splitting in Staphylococcus aureus is controlled by an adaptor protein facilitating degradation of a peptidoglycan hydrolase
Source: PLoS Genet. 2025 Sep 5;21(9):e1011841. doi: 10.1371/journal.pgen.1011841 (PMC12443321; doi:10.1371/journal.pgen.1011841)

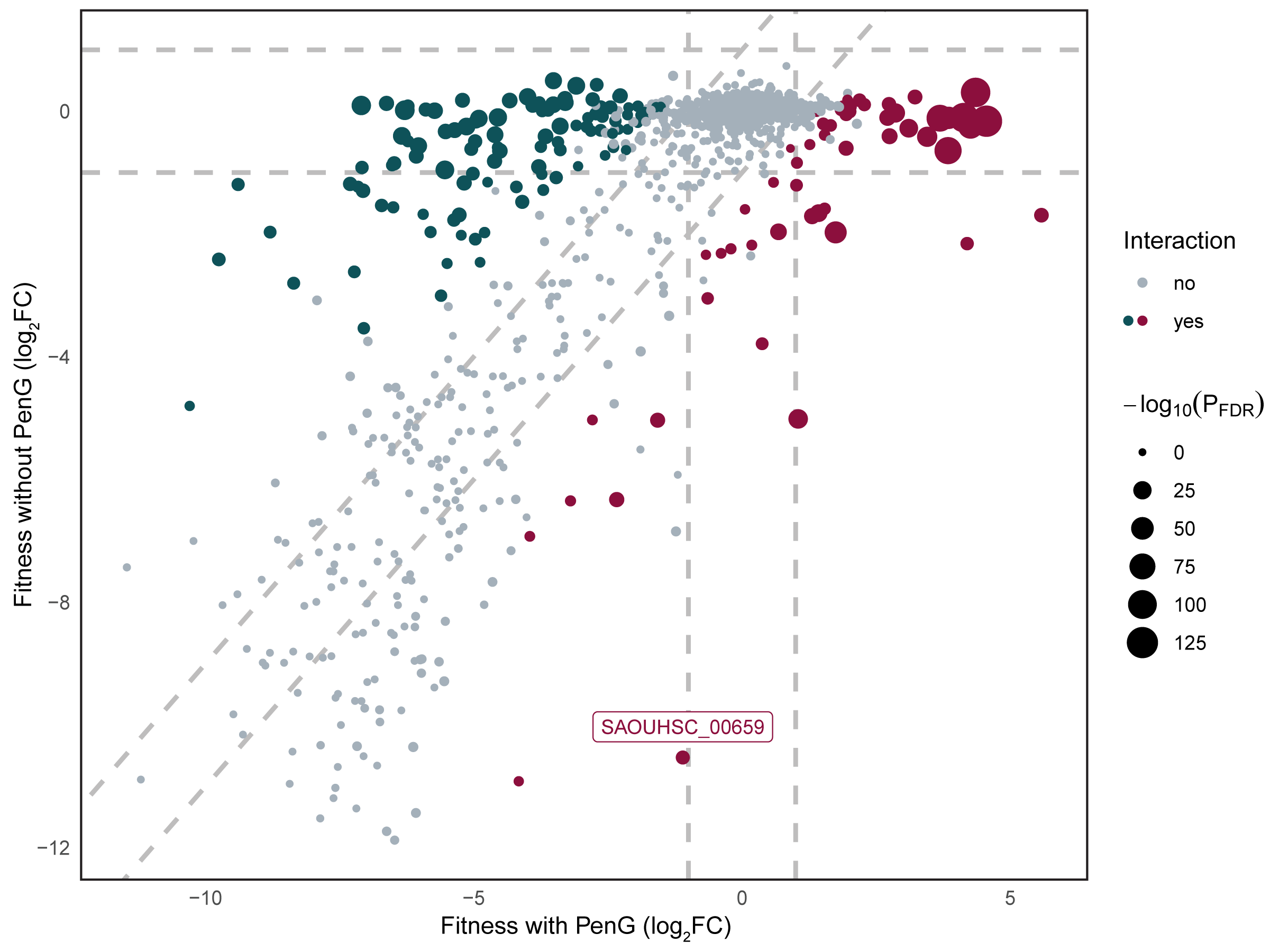

Supplement: S1 Fig — The growth fitness impact of knocking down each transcriptional unit was assessed under conditions with (x-axis) and without (y-axis) penicillin G (0.008 µg/mL). sgRNAs found to be significantly reduced upon penicillin G treatment, indicating that knockdown of these transcriptional units increases sensitivity to penicillin G, are highlighted in blue (log2FC < -1, Padj < 0.05). In contrast, sgRNAs that were significantly enriched, indicating that their knockdown reduces sensitivity to penicillin G, are highlighted in red (log2FC > 1, Padj < 0.05). Among the significantly enriched sgRNA, SAOUHSC_00659 (cxaR) was the top hit. (TIF) [file pgen.1011841.s001.tif]

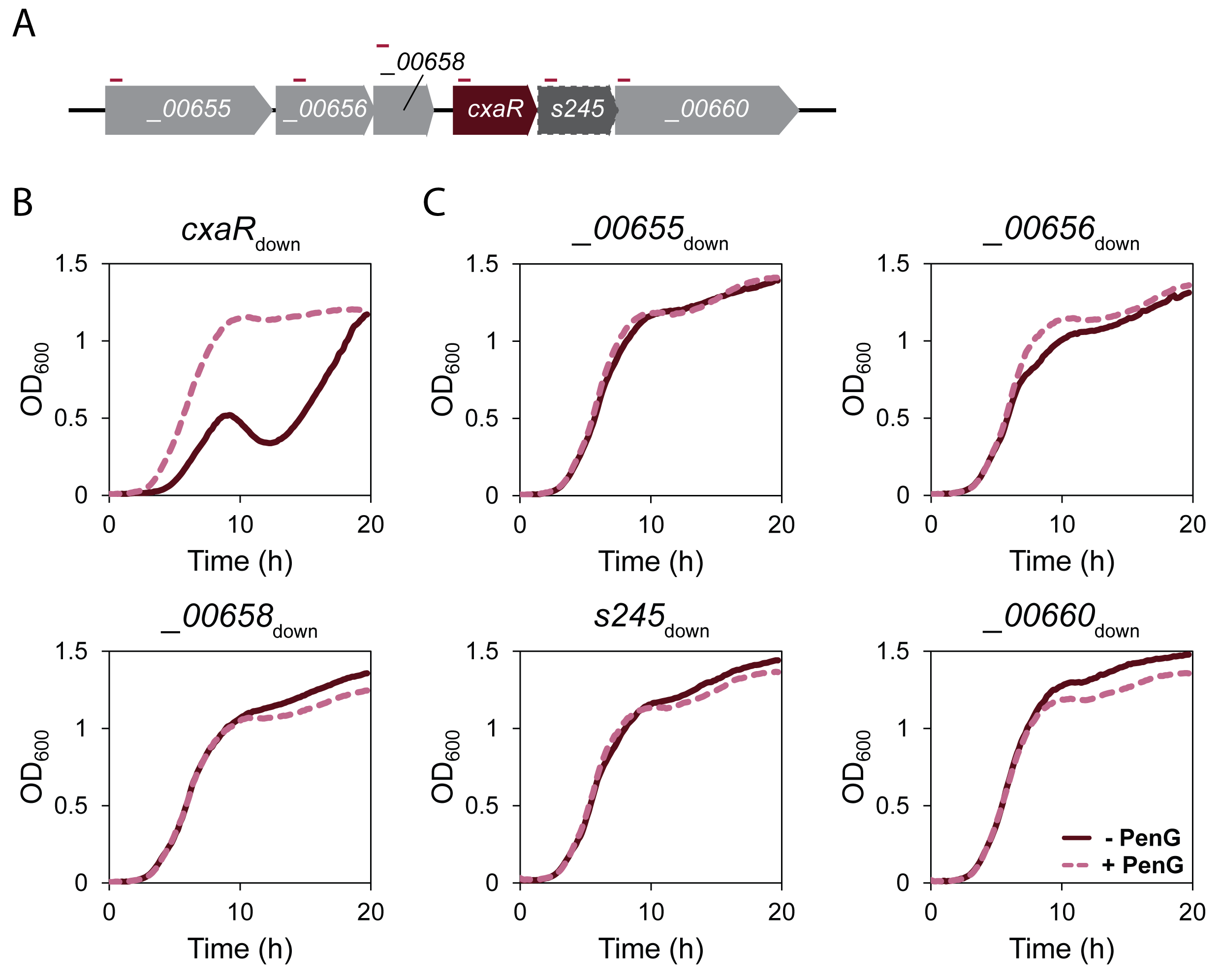

Supplement: S2 Fig — (A) Schematic overview of the genes surrounding cxaR in its operon. Red lines indicate the approximate location of sgRNA targets used for CRISPRi knockdown. (B) Growth curves of an NCTC8325–4 cxaRdown strain in TSB at 37°C with (dashed line) and without (solid line) 1 ng/mL of penicillin G. Knockdown was induced by 30 ng/mL aTc. (C) Growth curves showing the effects of CRISPRi knockdown of genes in NCTC8325–4 located upstream (SAOUHSC_00655, SAOUHSC_00656 and SAOUHSC_00658) and downstream (S245 and SAOUHSC_00660) of cxaR, with (dashed line) and without (solid line) 1 ng/mL of penicillin G. The strains were grown at 37°C in TSB with 30 ng/mL aTc to induce knockdown. (TIF) [file pgen.1011841.s002.tif]

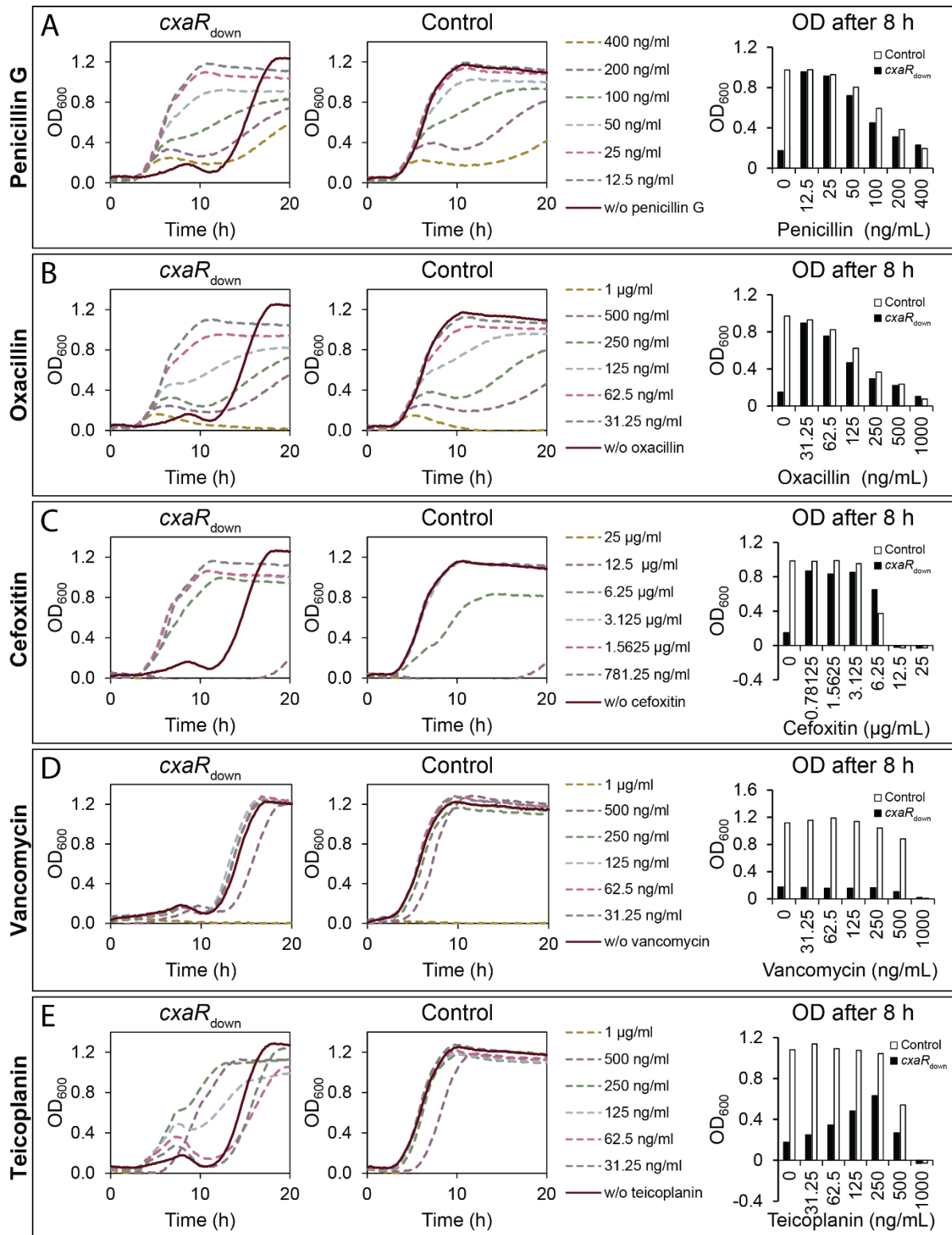

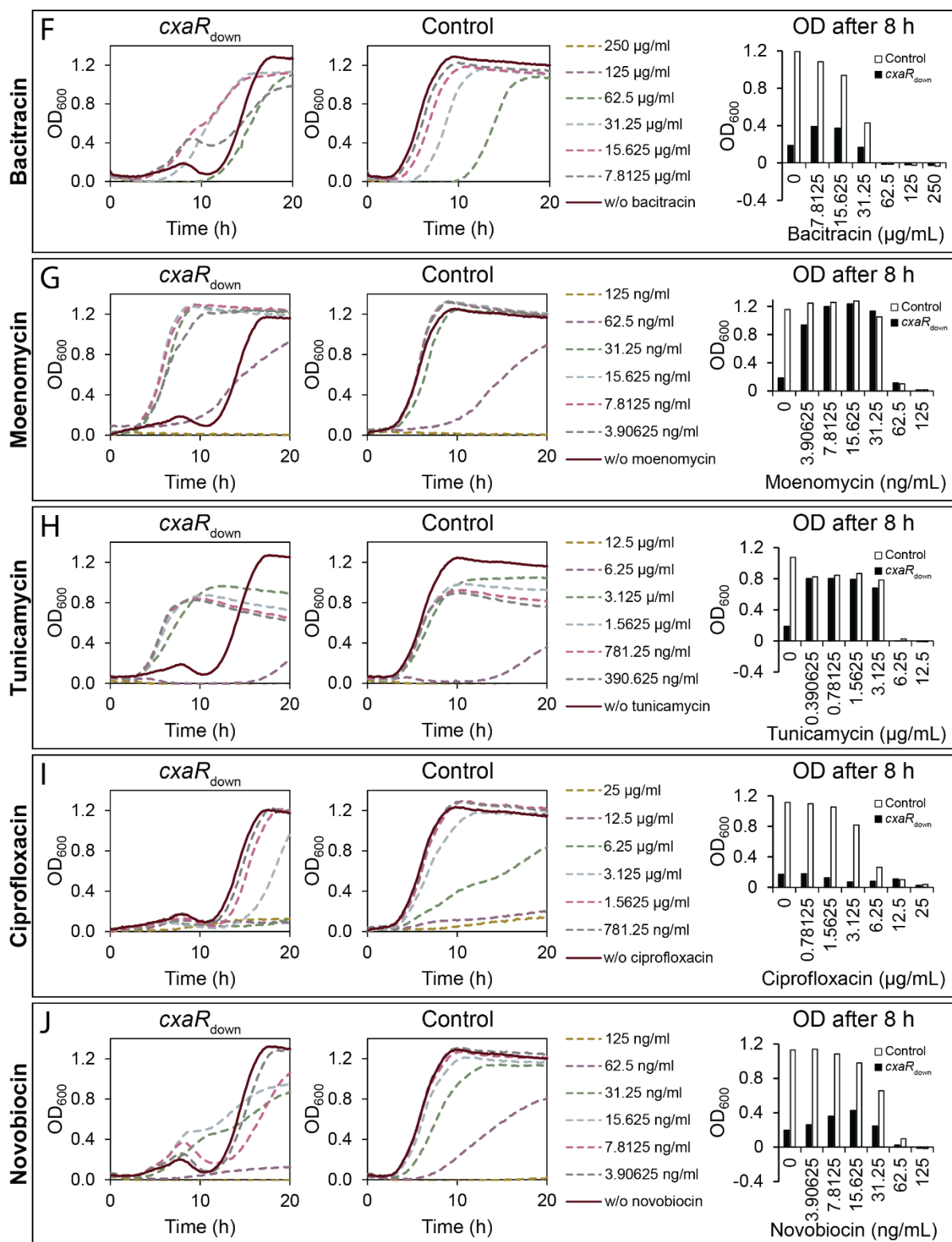

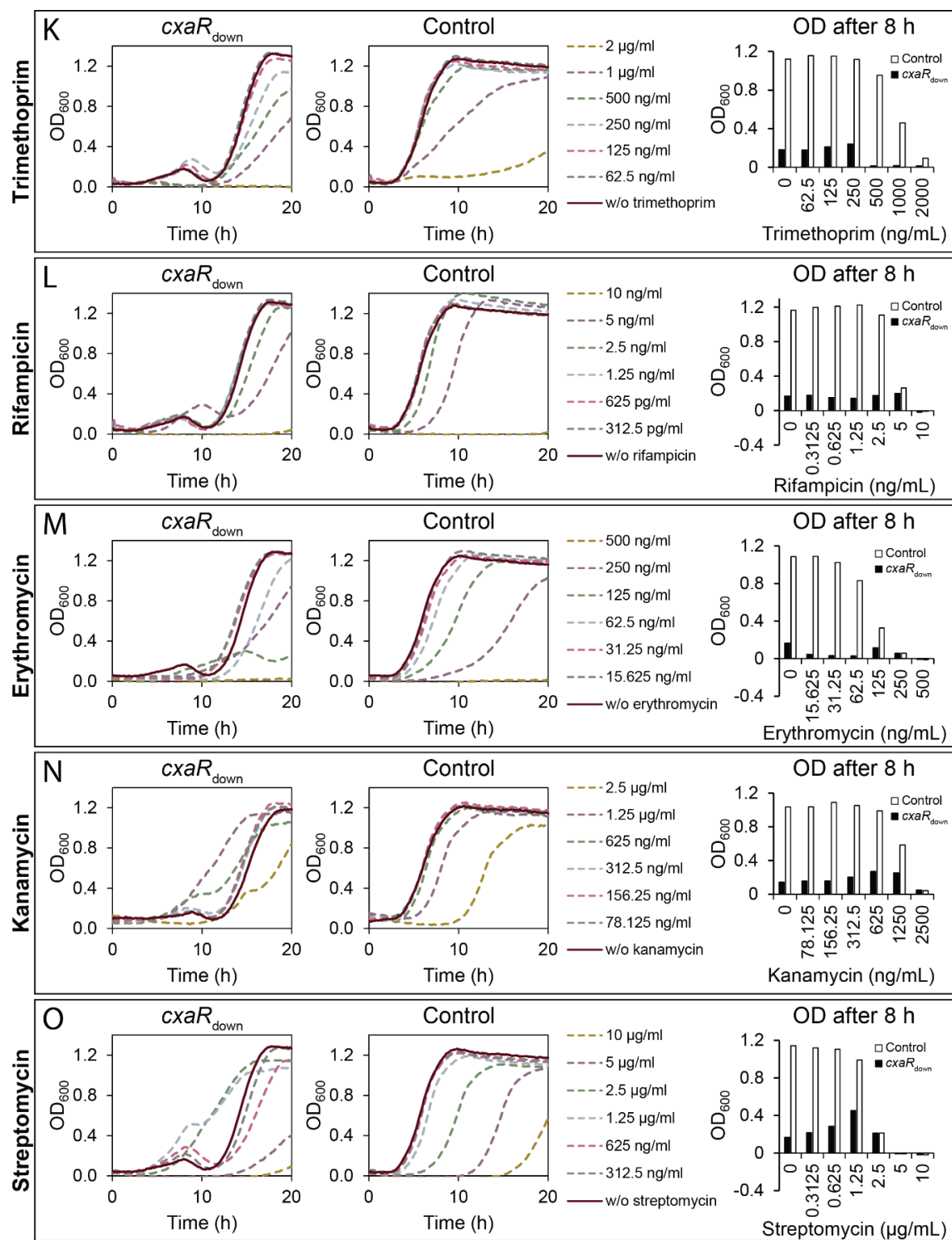

Supplement: S3 Fig — The JE2 cxaRdown strain (left panels) and the CRISPRi control (middle panels) subjected to various concentrations of different antibiotics during growth in TSB at 37°C. Knockdown was induced by 500 µM IPTG. The antibiotics tested included the β-lactams (A) penicillin G, (B) oxacillin, and (C) cefoxitin, the glycopeptides (D) vancomycin and (E) teicoplanin, the polypeptide antibiotic (F) bacitracin, the phosphoglycolipid antibiotic (G) moenomycin, the WTA-inhibiting nucleoside antibiotic (H) tunicamycin, the fluoroquinolone (I) ciprofloxacin, the aminocoumarin (J) novobiocin, the folate biosynthesis inhibitor (K) trimethoprim, the ansamycin (L) rifampicin, the macrolide (M) erythromycin, and the aminoglycosides (N) kanamycin and (O) streptomycin. The right panels show the OD600 for each strain after 8 hours of growth for each concentration of the antibiotics tested. (PDF) [file pgen.1011841.s003.pdf]

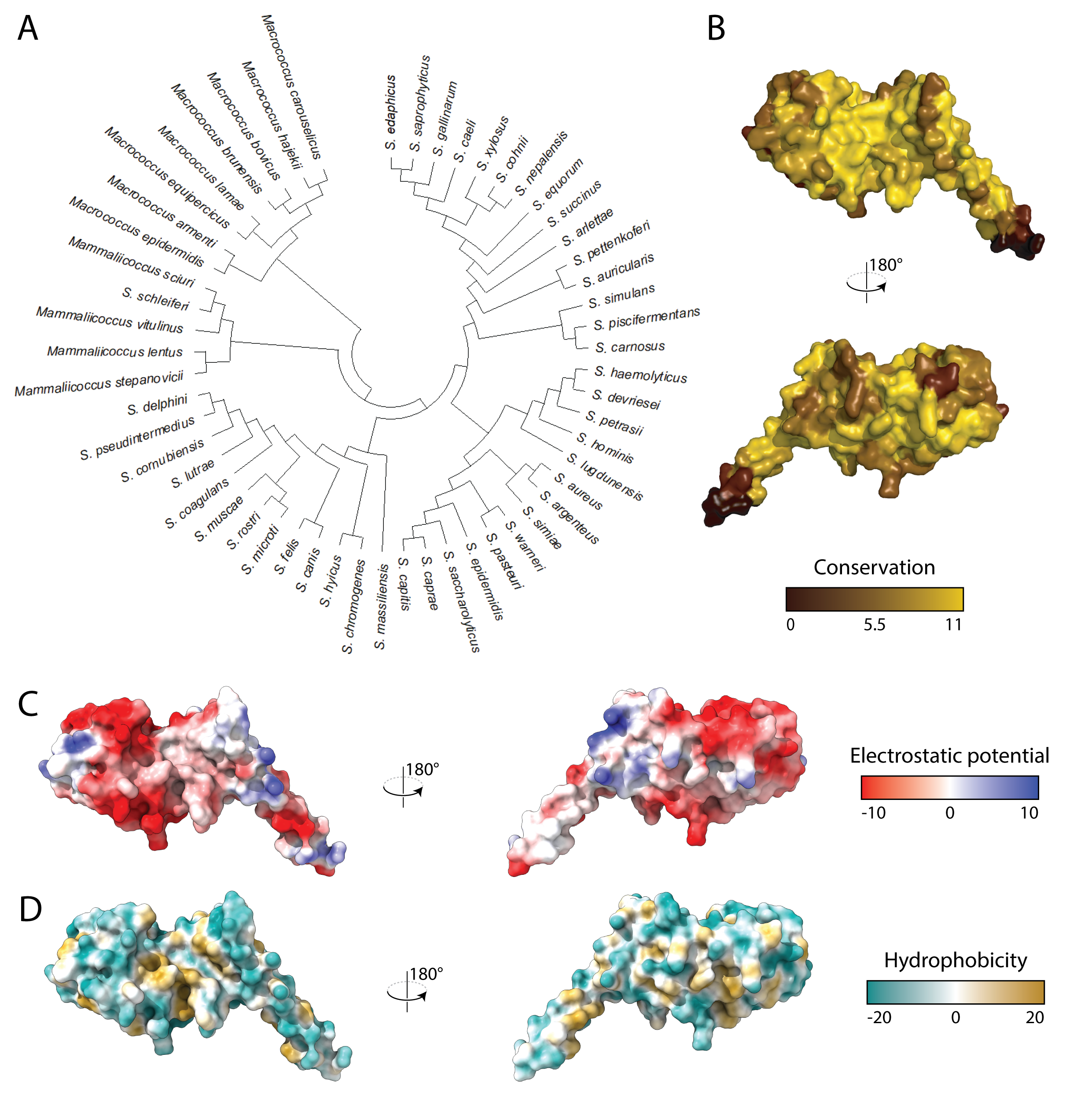

Supplement: S4 Fig — (A) Phylogenetic tree of CxaR homologues from 55 species within the Staphylococcaceae family. The phylogenetic relatedness of the homologues generally clustered by genera, with the exception of Staphylococcus schleiferi which appeared to utilize a CxaR homolog more closely resembling those found in Mammaliicoccus. (B) AlphaFold 3-predicted structure of CxaR with residue conservation from the phylogenetic analysis in A mapped on to it. The colors represent conservation scores ranging from 0 (least conserved) to 11 (most conserved), accounting for both conservation of specific amino acids and their stereochemical properties. (C) Coulombic electrostatic potential of the predicted CxaR structure, with red for negative potential through white to blue for positive potential. (D) Hydrophobicity (molecular lipophilicity potential) of the predicted CxaR structure, with dark cyan for most hydrophilic through white to dark goldenrod for most hydrophobic. (TIF) [file pgen.1011841.s004.tif]

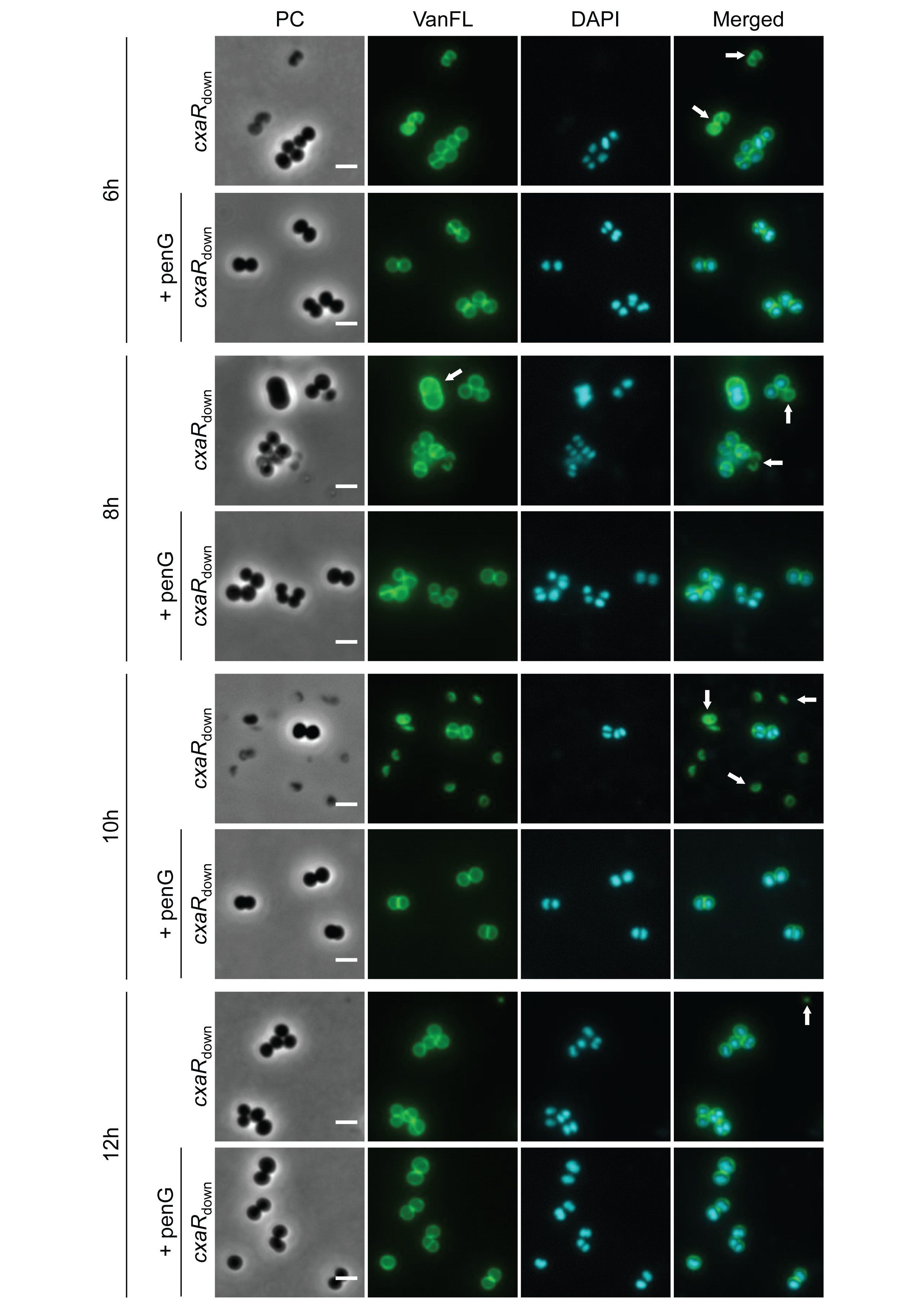

Supplement: S5 Fig — Phase contrast and fluorescence microscopy of the JE2 cxaRdown strain after 6, 8, 10, and 12 hours of incubation in TSB at 37°C, with and without 5 ng/mL penicillin G. CRISPRi knockdown of cxaR expression was induced by adding IPTG to a final concentration of 500 µM. The cells were stained with VanFL (cell wall) and DAPI (nucleoid). White arrows indicate abnormal and recently lysed cells. Scale bars represent 2 µm. (TIF) [file pgen.1011841.s005.tif]

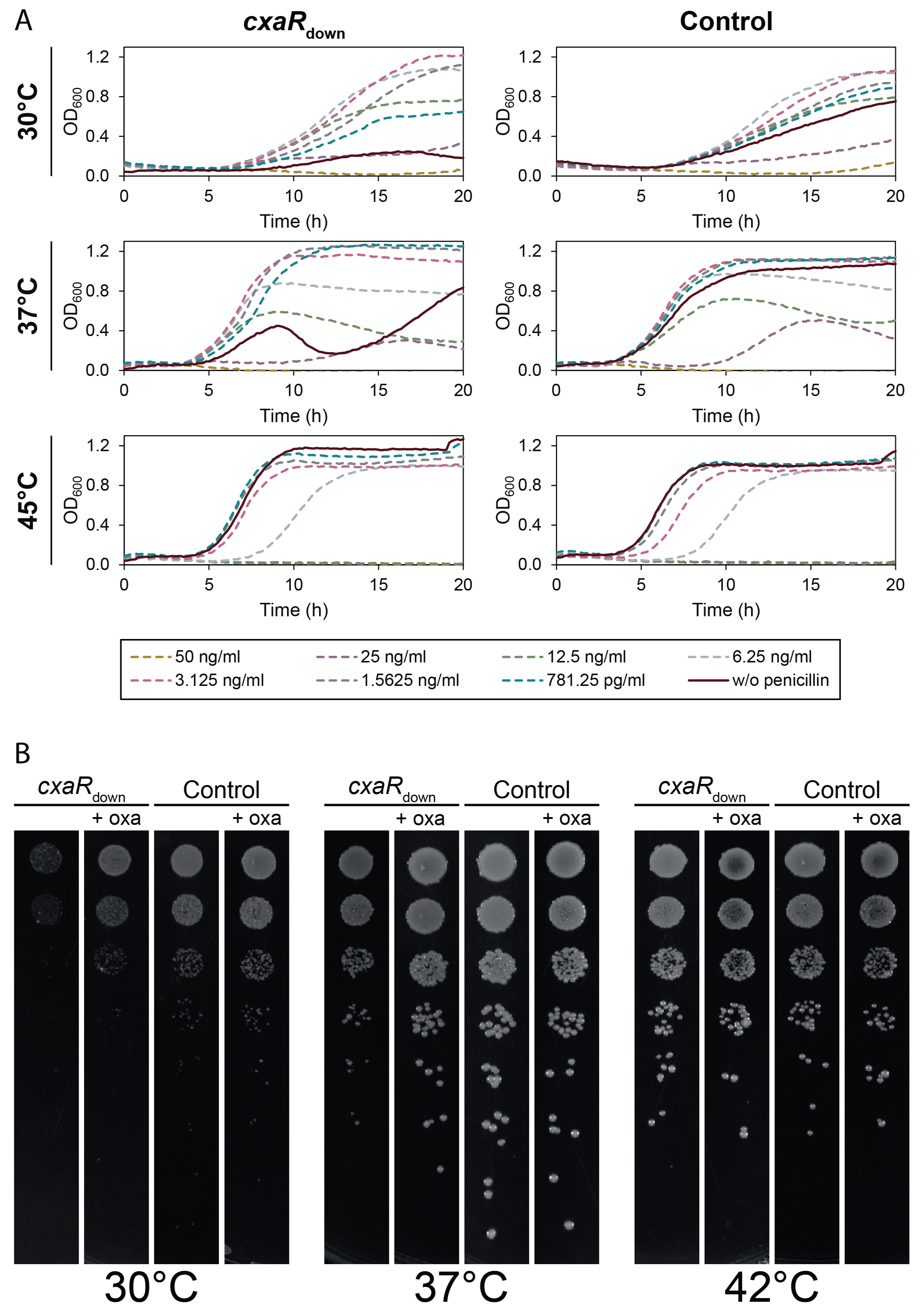

Supplement: S6 Fig — (A) The NCTC8325–4 cxaRdown and CRISPRi control strains grown in TSB at 30°C, 37°C, and 45°C with different concentrations of penicillin G. Gene knockdown was induced with the addition of 500 μM IPTG. The graphs represent averages from duplicate measurements. (B) Spotting of the NCTC8325–4 cxaRdown and CRISPRi control strains at different temperatures with or without addition on oxacillin. The strains were grown until an OD600 of 0.5 was reached, serially diluted in 10-fold dilution series, and 5 µL of each dilution was spotted on TSA containing 500 μM IPTG, with or without 6 ng/mL oxacillin. The plates were incubated at 30°C, 37°C, or 42°C for 24 hours before imaging. (TIF) [file pgen.1011841.s006.tif]

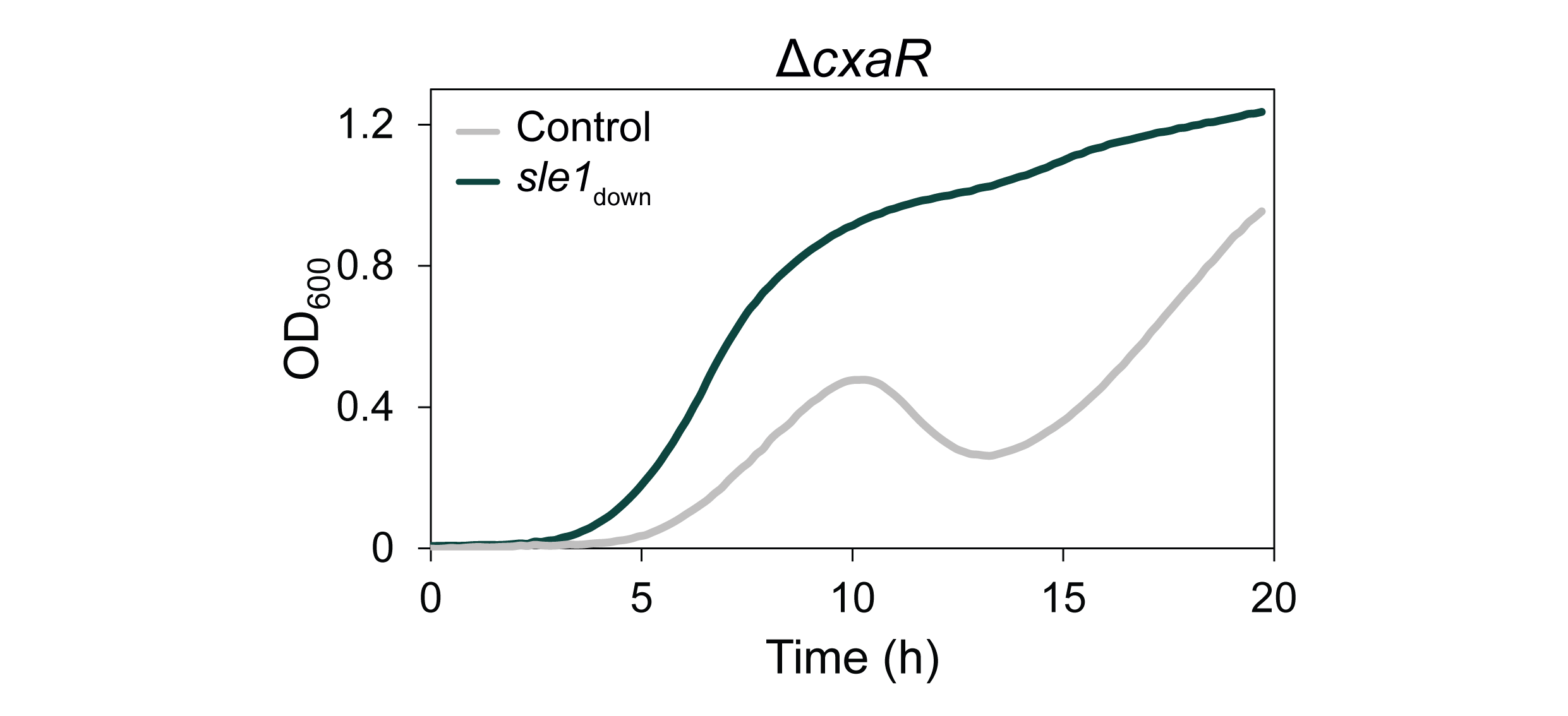

Supplement: S7 Fig — Growth of an NCTC8325–4 ΔcxaR mutant with (green) and without (grey) knockdown of sle1 in TSB at 37°C. Gene knockdown was induced with the addition of 500 μM IPTG. The graphs represent averages from triplicate measurements. (TIF) [file pgen.1011841.s007.tif]

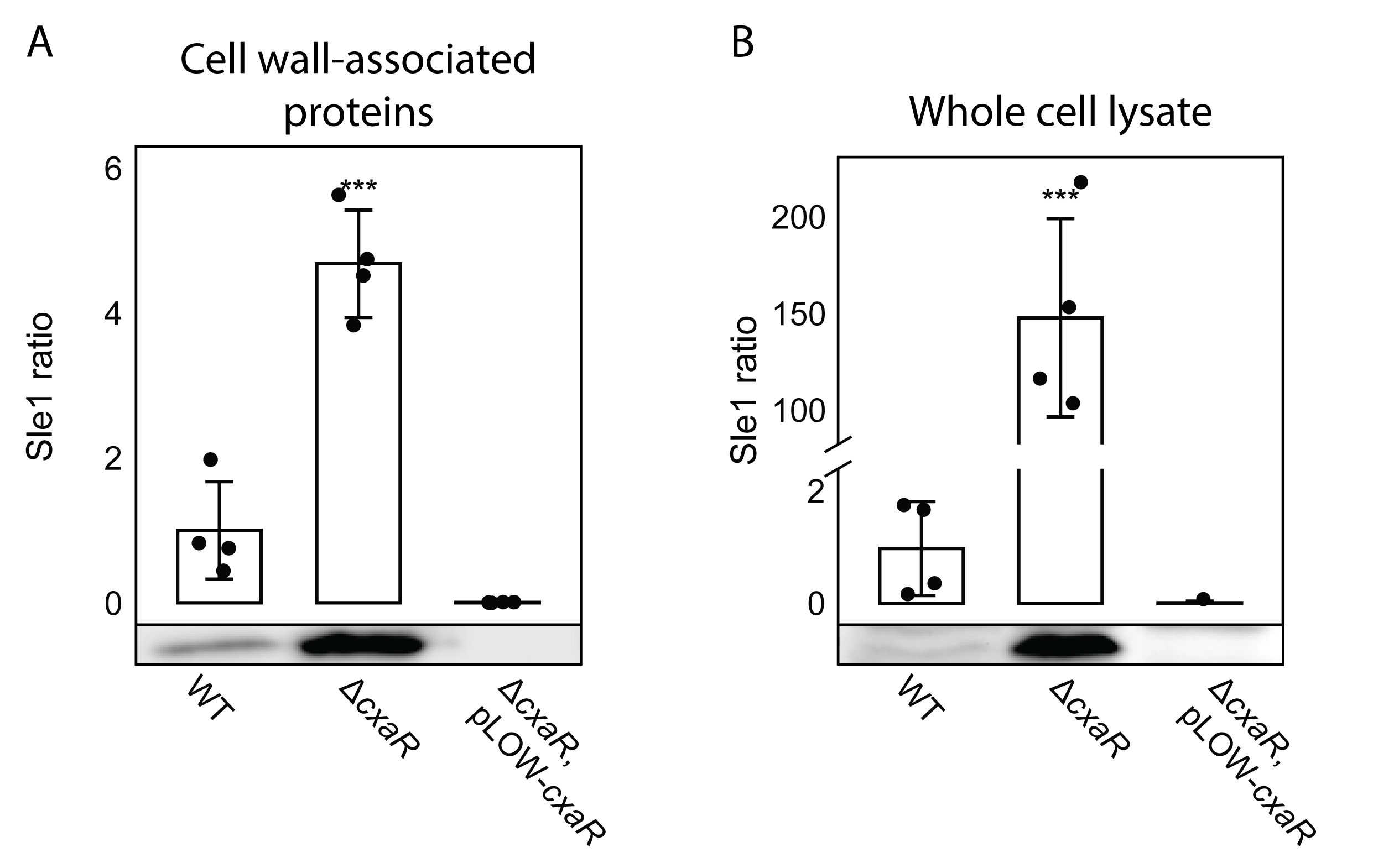

Supplement: S8 Fig — Western blot analysis of Sle1 levels in (A) cell wall-associated protein fractions and (B) whole-cell lysates from NCTC8325–4 wild-type, cxaR deletion, and cxaR overexpressing cells. Band intensities were quantified using ImageJ and normalized to the wild-type signal. (A) In cell wall-associated fractions, Sle1 levels were increased in ΔcxaR cells (4.681 ± 1.270) and reduced in CxaR overexpressing cells (0.007 ± 0.003), relative to the wild-type (1.000 ± 0.220). (B) Similarly, in whole-cell lysates, Sle1 levels were highly elevated in ΔcxaR cells (147.847 ± 45.768) and depleted in CxaR overexpressing cells (0.015 ± 0.043), compared to the wild-type (1.000 ± 1.095). Statistical significance was assessed using one-way ANOVA followed by Tukey’s Honest Significant Difference (HSD) test (***, P-value < 0.001). Column height represents the mean, and error bars the standard deviation from four biological replicates. Representative Sle1 blots are shown below the corresponding graphs. (TIF) [file pgen.1011841.s008.tif]

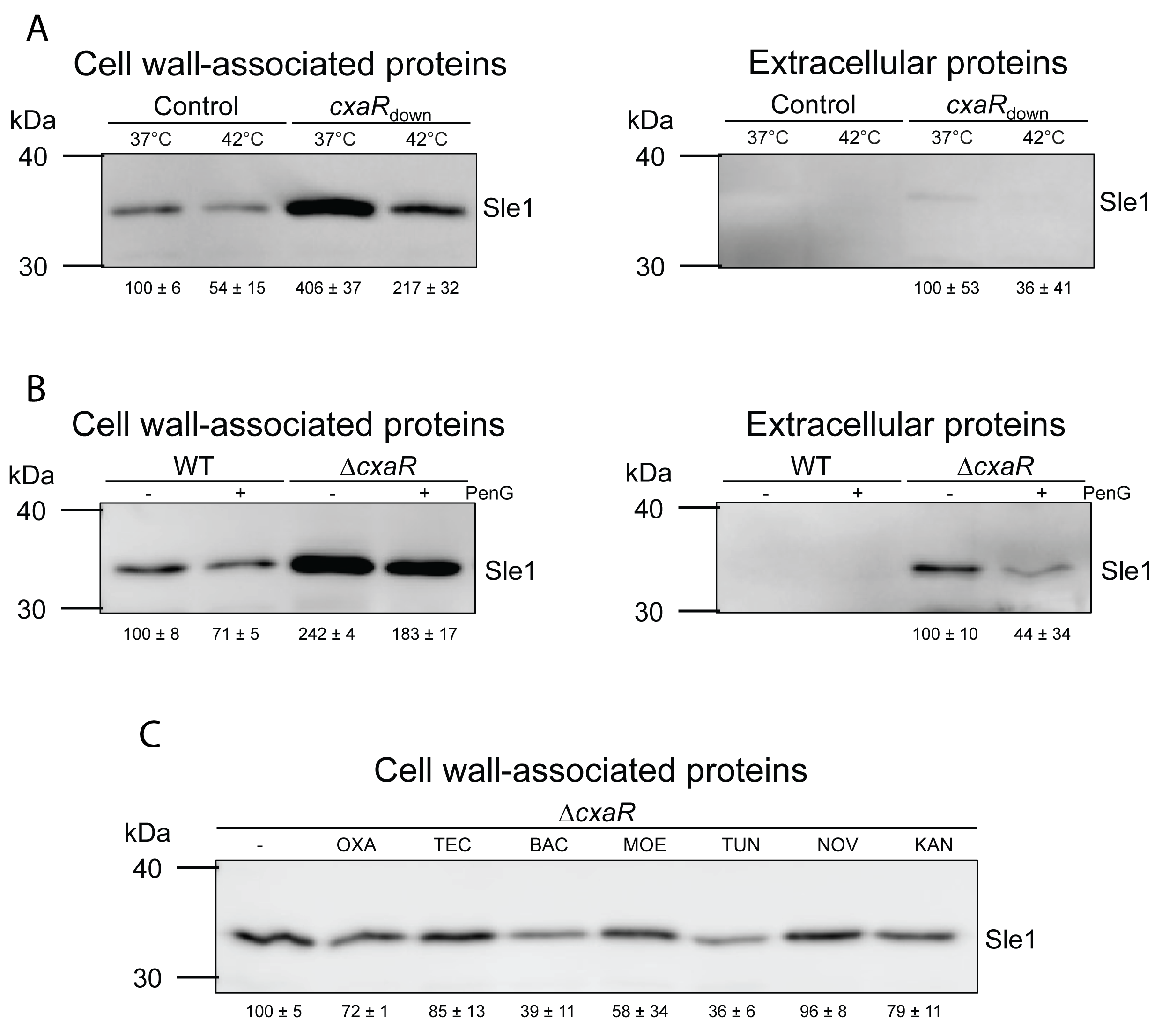

Supplement: S9 Fig — (A) Western blots displaying the difference in cell wall-associated and extracellular Sle1 levels in the NCTC8325–4 cxaRdown strain and CRISPRi control incubated at 37°C and 42°C. IPTG (500 μM) was added for gene knockdown. Band intensities were quantified using ImageJ. Values for cell wall-associated Sle1 were normalized to the control band at 37 °C, while extracellular Sle1 values were normalized to the cxaRdown band at 37 °C. The intensities, shown below the corresponding bands, represent the mean of two biological replicates, with standard deviation. (B) The effect of sublethal concentrations of penicillin G (1 ng/mL) on the cell wall-associated and extracellular levels of Sle1 in the NCTC8325–4 wild-type and ΔcxaR mutant, shown by Western blotting. Band intensities were quantified using ImageJ. Values for cell wall-associated Sle1 were normalized to the wild-type band without penicillin G, while extracellular Sle1 values were normalized to the ΔcxaR band without penicillin G. The intensities, shown below the corresponding bands, represent the mean of two biological replicates, with standard deviation. (C) Western blotting of cell wall-associated Sle1 in the NCTC8325–4 ΔcxaR mutant in response to sublethal concentrations of various antibiotics. From left to right; - (without antibiotics), OXA (7 ng/mL oxacillin), TEC (62.5 ng/mL teicoplanin), BAC (1 μg/mL bacitracin), MOE (7.5 ng/mL moenomycin), TUN (200 ng/mL tunicamycin), NOV (15 ng/mL novobiocin), KAN (300 ng/mL kanamycin). Band intensities were quantified using ImageJ. Values for cell wall-associated Sle1 were normalized to the ΔcxaR band without antibiotics. The intensities, shown below the corresponding bands, represent the mean of two biological replicates, with standard deviation. (TIF) [file pgen.1011841.s009.tif]

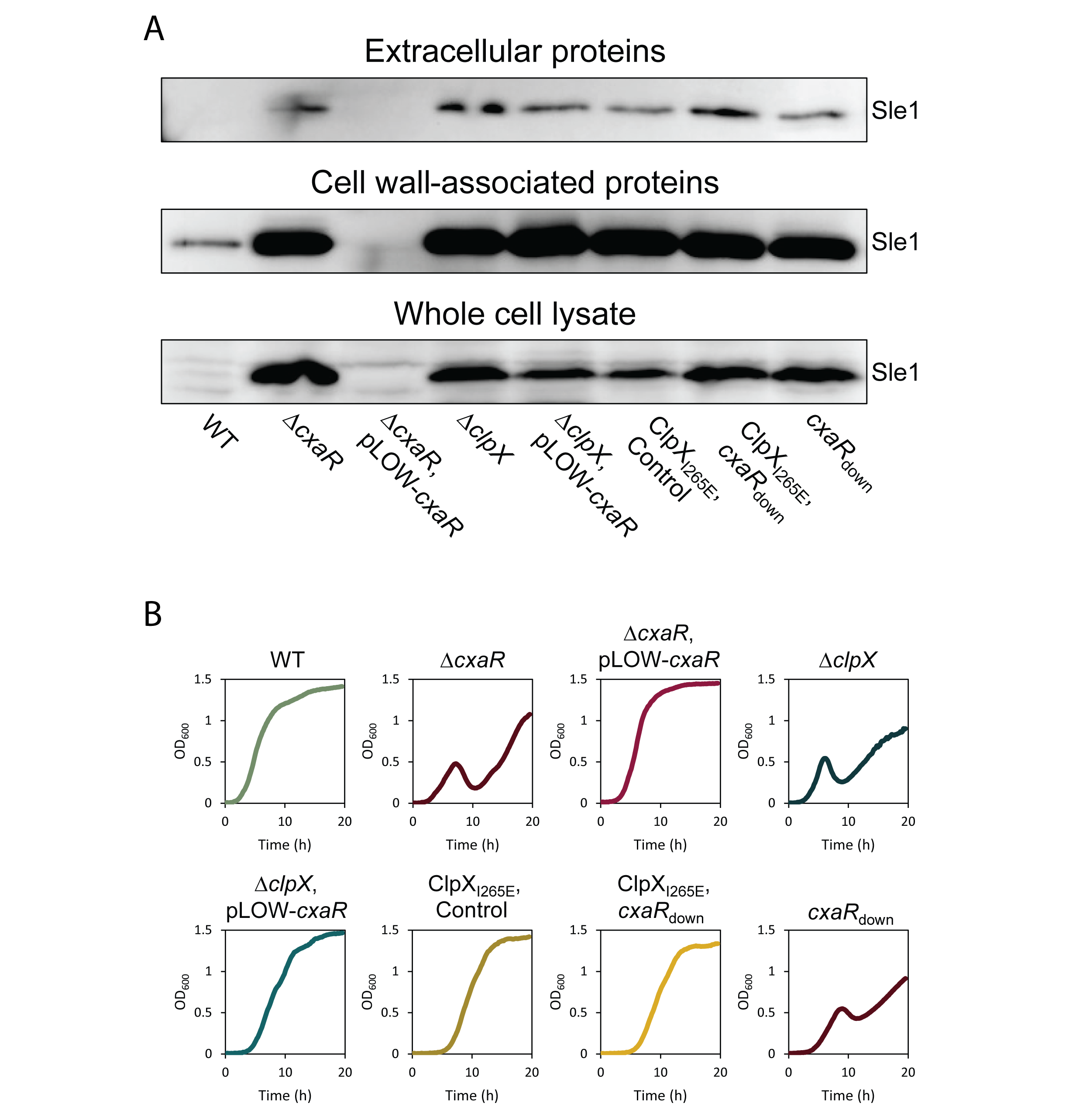

Supplement: S10 Fig — (A) Western blots of extracellular, cell wall-associated, and whole cell Sle1 levels in the NCTC8325–4 wildtype, ΔcxaR mutant, cxaR overexpressing strain, ΔclpX mutant, ΔclpX mutant overexpressing cxaR, clpXI265E mutant, clpXI265E mutant with knockdown of cxaR, and cxaR knockdown strain. Gene knockdown and overexpression were induced with the addition of 500 μM IPTG. (B) Growth curves displaying the growth phenotypes of the strains Western blotted in A at 37°C in TSB. Gene knockdown and overexpression were induced with the addition of 500 μM IPTG. The graphs represent averages from triplicate measurements. (TIF) [file pgen.1011841.s010.tif]

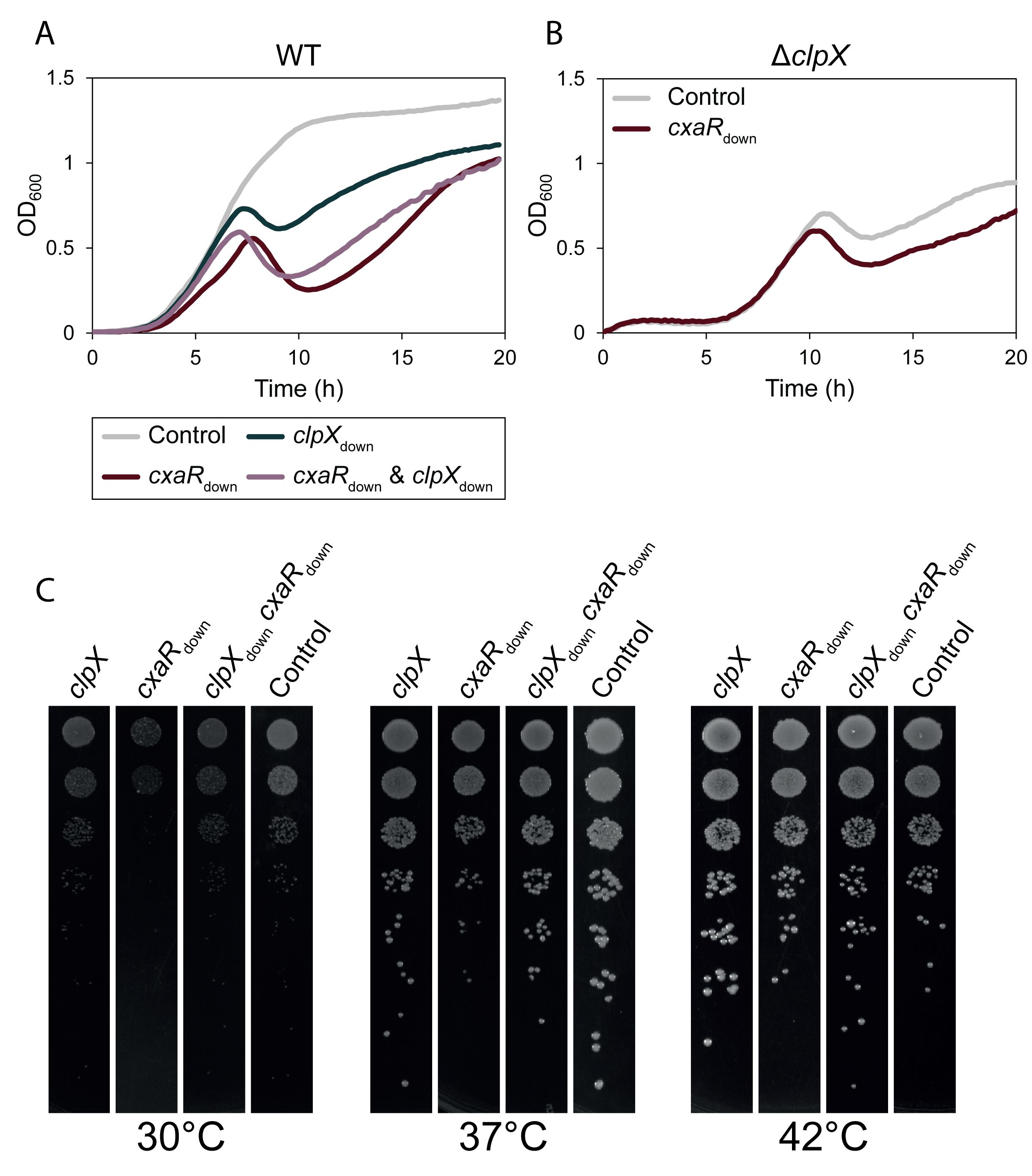

Supplement: S11 Fig — (A) The effect of concurrent CRISPRi knockdown of clpX and cxaR (purple) in NCTC8325–4 compared to the CRISPRi control (grey) and single knockdown of clpX (green) or cxaR (red) during growth in TSB at 37°C. Gene knockdown was induced with addition of 500 μM IPTG. The graphs represent averages from triplicate measurements. (B) Growth of an NCTC8325–4 ΔclpX mutant in TSB at 37°C with (red) and without (grey) knockdown of cxaR. Gene knockdown was induced with addition of 500 μM IPTG. The graphs represent averages from triplicate measurements. (C) Spotting assay of cells with single knockdown of either clpX (clpXdown) or cxaR (cxaRdown), and double knockdown of both clpX and cxaR (clpXdown cxaRdown) compared to the CRISPRi control at 30°C, 37°C, and 42°C. The strains were grown to mid-exponential phase (OD600 of 0.5), 10-fold serially diluted, and spotted in volumes of 5 µL on TSA with 500 μM IPTG. (TIF) [file pgen.1011841.s011.tif]

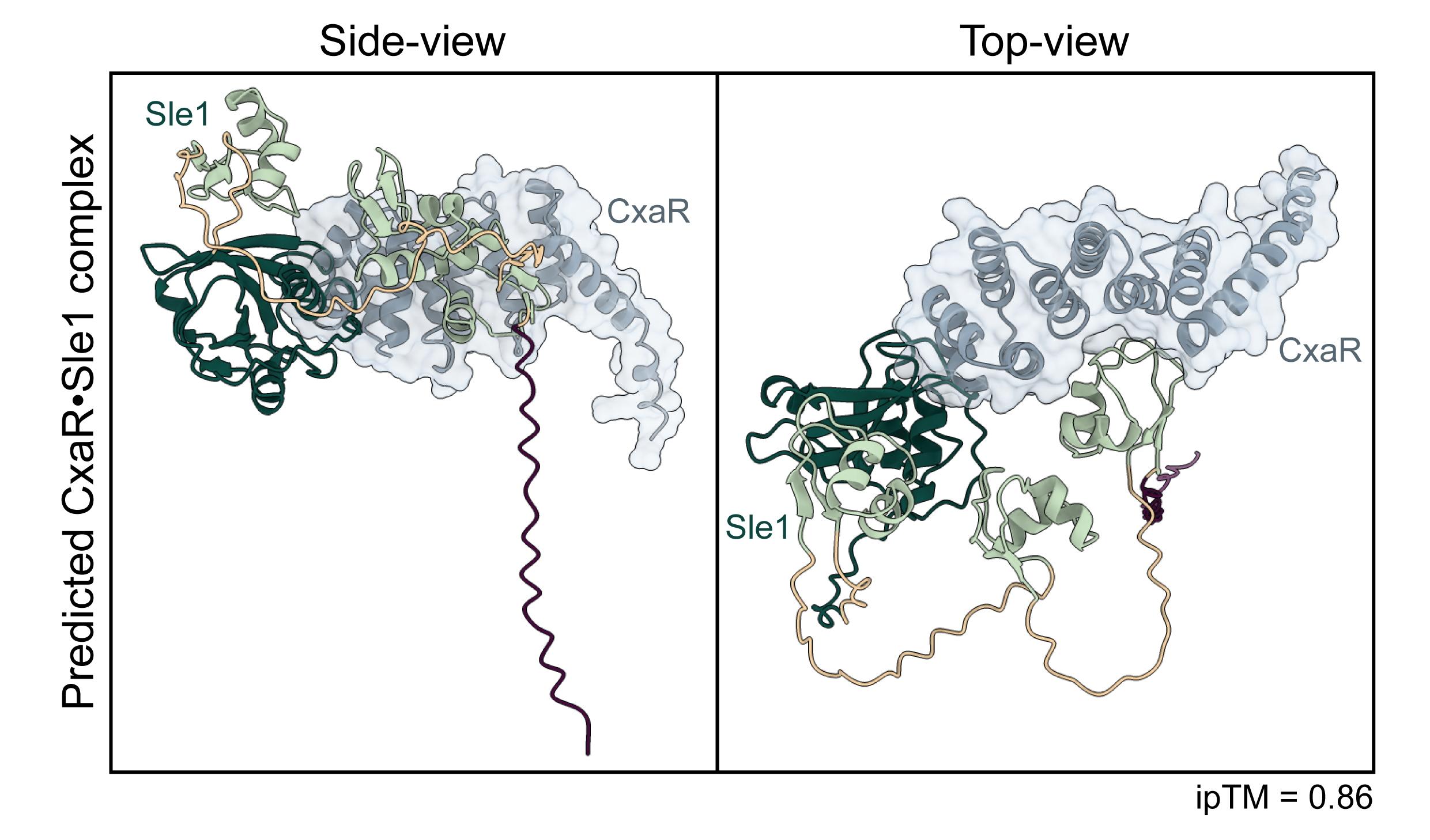

Supplement: S12 Fig — The domains of Sle1 are colored in accordance with the structure in Fig 6D, with the signal peptide in purple, LysM domains in light green, and CHAP domain in dark green. The predicted complex has a high confidence interaction score (ipTM) of 0.86. (TIF) [file pgen.1011841.s012.tif]

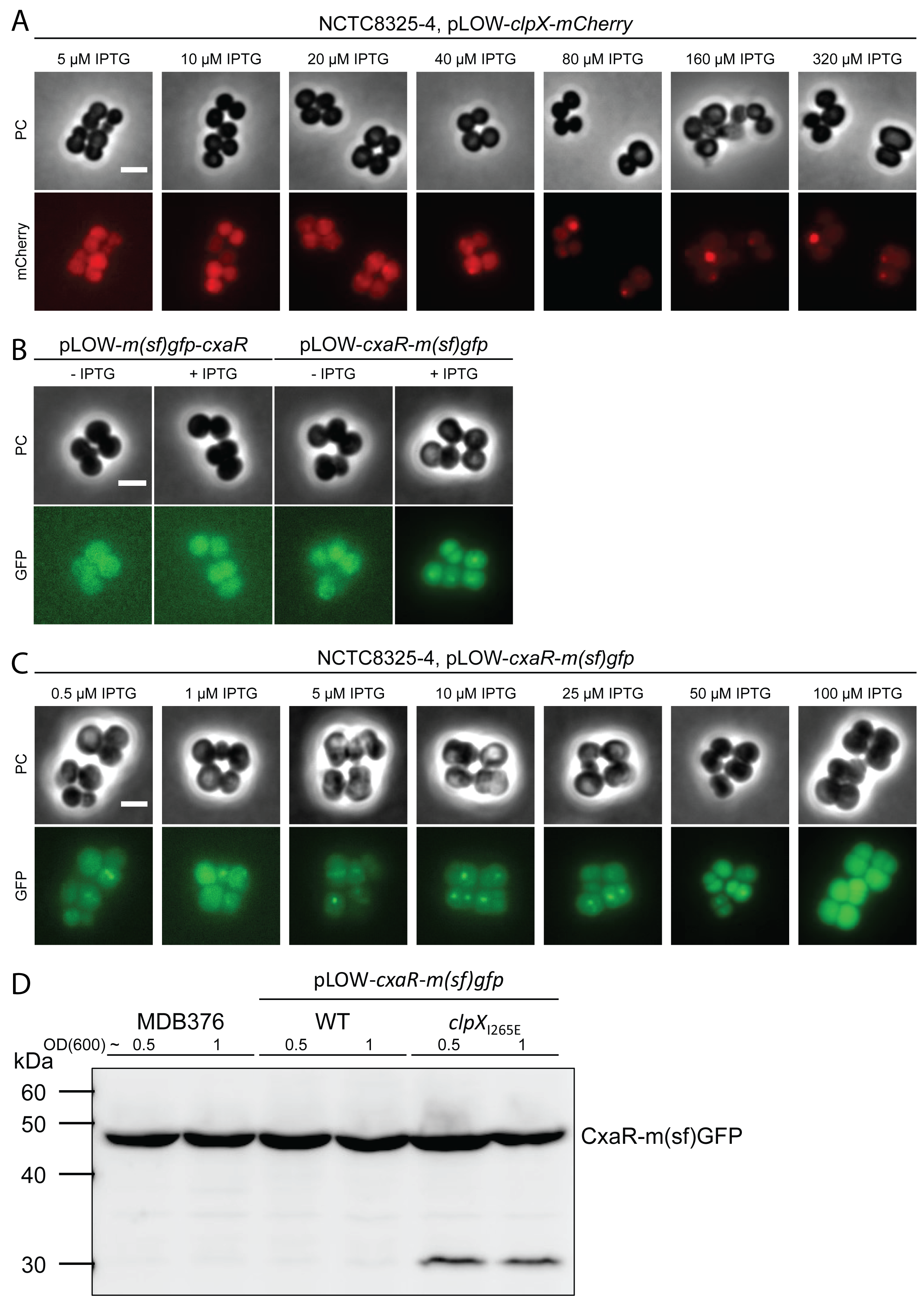

Supplement: S13 Fig — (A) Phase contrast and fluorescence microscopy of a plasmid-borne C-terminal ClpX-mCherry fusion in NCTC8325–4, with inducer concentrations between 5 µM and 320 µM IPTG. Scale bar represents 2 µm. (B) Phase contrast and fluorescence microscopy of NCTC8325–4 carrying plasmids encoding an N-terminal (left) and C-terminal (right) CxaR-GFP fusion, with and without induction of expression by 50 µM IPTG. Scale bar represents 2 µm. (C) Various induction levels of the plasmid-borne C-terminal CxaR-GFP fusion in NCTC8325–4. The concentrations of the IPTG inducer ranged from 0.5 µM to 100 µM, and localization was assessed with phase contrast and fluorescence microscopy. Scale bar represents 2 µm. (D) Western blot of the C-terminal CxaR-GFP fusion from whole cell lysate expressed from the native cxaR chromosomal locus in NCTC8325–4 (MDB376), from the pLOW plasmid in the NCTC8325–4 wild-type and from the pLOW plasmid in the NCTC8325–4 clpXI265E mutant. Expression of the fusion from pLOW was induced by 10 µM IPTG. Cultures were harvested at OD600 0.5 and 1.0. (TIF) [file pgen.1011841.s013.tif]

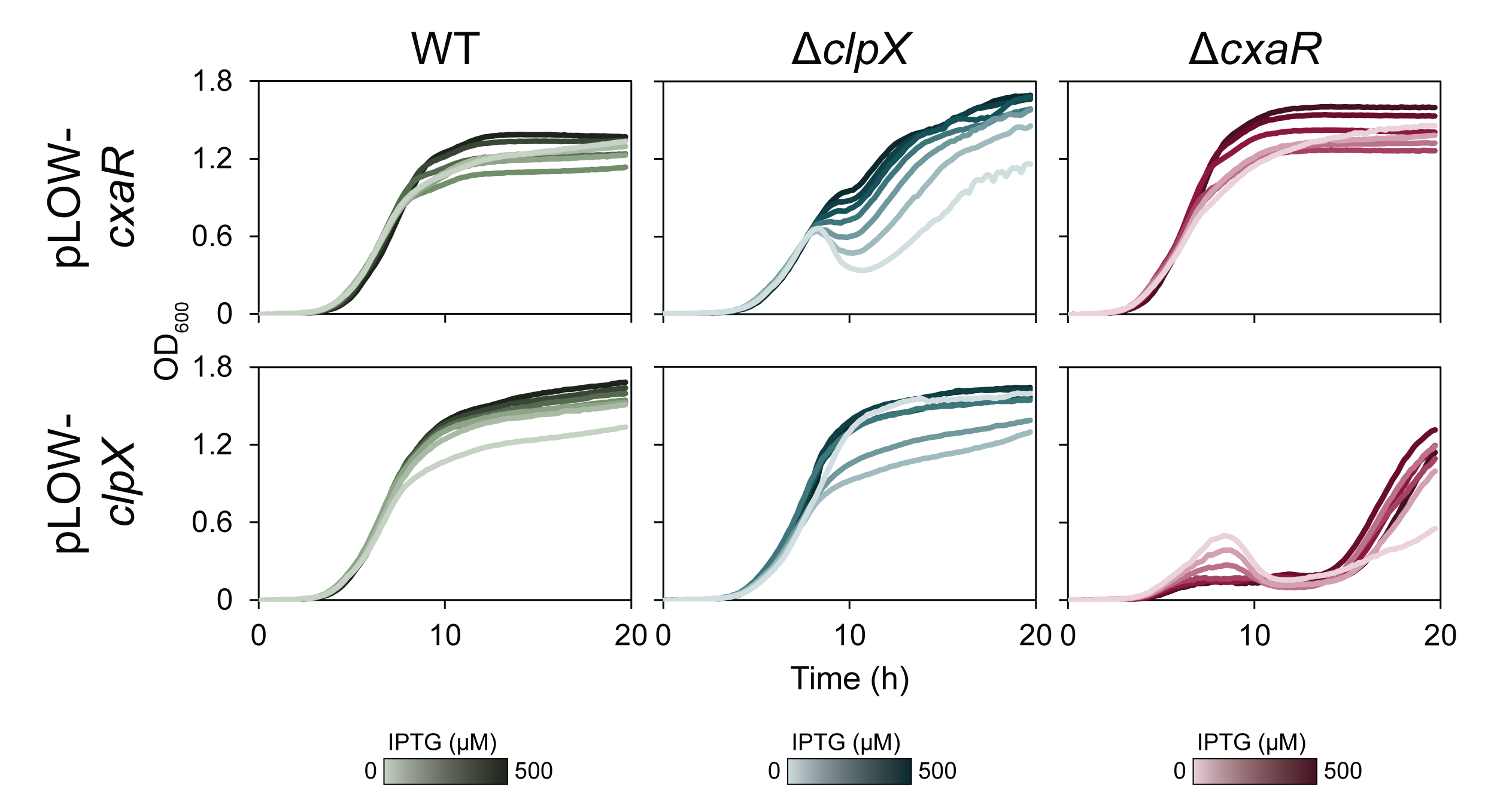

Supplement: S14 Fig — Overexpression of cxaR and clpX in the NCTC8325–4 wild-type (green), ΔclpX mutant (blue) and ΔcxaR mutant (red) from pLOW-cxaR and pLOW-clpX, respectively. Concentrations of IPTG ranged from 0 to 500 µM. (TIF) [file pgen.1011841.s014.tif]
