# Supplementary material for: Cell splitting in Staphylococcus aureus is controlled by an adaptor protein facilitating degradation of a peptidoglycan hydrolase
Source: PLoS Genet. 2025 Sep 5;21(9):e1011841. doi: 10.1371/journal.pgen.1011841 (PMC12443321; doi:10.1371/journal.pgen.1011841)
Supplement: S2 Table — List of mutations, with over 90% variant frequency, identified when re-sequencing five independently isolated, visibly larger NCTC8325–4 ΔcxaR colonies formed at 30°C. (PDF) [file pgen.1011841.s016.pdf]

S2 Table. The spontaneous mutations acquired when the *cxaR* deletion mutant was grown at a 30°C. List of mutations, with over 90% variant frequency, identified when re-sequencing five independently isolated, visibly larger NCTC8325-4 Δ*cxaR* colonies formed at 30°C.

Strain name	Gene affected	Nucleotide change	Polymorphism type	Amino acid change	Protein effect	Variant Frequency
EFS104	*aldA*	G>T	SNP (transversion)	E375D	Substitution	99.5 %
	(RBS of *sle1*)	G>A (at position −13 relative to the *sle1* start codon)	SNP (transition)			99.3 %
EFS105	*sle1*	G>A	SNP (transition)	M292I	Substitution	99.7 %
MDB390	*sle1*	G>T	SNP (transversion)	G315C	Substitution	100.0%
MDB401	*sle1*	C>T	SNP (transition)	Q197*	Truncation	99.7%
	sarX	C>T	SNP (transition)	H65Y	Substitution	100.0%
MDB404	*sle1*	(A)8>(A)7	Deletion (tandem repeat)	V5*	Frame Shift	99.7%
