# Supplementary material for: Cell splitting in Staphylococcus aureus is controlled by an adaptor protein facilitating degradation of a peptidoglycan hydrolase
Source: PLoS Genet. 2025 Sep 5;21(9):e1011841. doi: 10.1371/journal.pgen.1011841 (PMC12443321; doi:10.1371/journal.pgen.1011841)
Supplement: S3 Table — (PDF) [file pgen.1011841.s017.pdf]

S3 Table. Strains and mutants used in this work.

Name	Genotype and characteristics[a]	Reference
***S. aureus* NCTC8325-4**		
NCTC8325-4/ MDB1	MSSA lab strain, derivative of NCTC8325 cured of prophages	[1]
MM267	NCTC8325-4, *tetR*_P_{tet}_*dCas9*, *tetM*	Lab collection
MHU16	MM267 carrying pVL2336-sgRNA(*cxaR*), cam[r]	This work
MHU46	MM267 carrying pVL2336-sgRNA(*S245*), cam[r]	This work
MHU48	MM267 carrying pVL2336-sgRNA(*SAOUHSC_00660*), cam[r]	This work
MHU57	MM267 carrying pVL2336-sgRNA(*SAOUHSC_00655*), cam[r]	This work
MHU58	MM267 carrying pVL2336-sgRNA(*SAOUHSC_00656*), cam[r]	This work
MHU59	MM267 carrying pVL2336-sgRNA(*SAOUHSC_00658*), cam[r]	This work
MK2047	NCTC8325-4, ∆*cxaR*, spc[r]	This work
EFS104	MK2047, *sle1* $RBS_{G\rightarrow A}$, spc[r]	This work
EFS105	MK2047, $sle1_{M292I}$, spc[r]	This work
MDB390	MK2047, $sle1_{G315C}$, spc[r]	This work
MDB401	MK2047, $sle1_{Q197*}$, spc[r]	This work
MDB404	MK2047, $sle1_{V5*}$, spc[r]	This work
MK2061	MK2047 carrying pLOW-*dCas9*, spc[r], ery[r]	This work
MK2063	MK2061 carrying pVL2236-sgRNA(*sle1*), spc[r], ery[r], cam[r]	This work
MK2066	MK2061 carrying pCG248-sgRNA(*luc*), spc[r], ery[r], cam[r]	This work
MDB432	MK2047 carrying pLOW-*cxaR*, spc[r], ery[r]	This work
MDB433	MK2047 carrying pLOW-*cxaR-m(sf)gfp*, spc[r], ery[r]	This work
MDB447	MK2047 carrying pLOW-*clpX-mCherry*, spc[r], ery[r]	This work
MDB429	NCTC8325-4 carrying pLOW-*cxaR*, ery[r]	This work
MDB430	NCTC8325-4 carrying pLOW-*cxaR-m(sf)gfp*, ery[r]	This work
MDB431	NCTC8325-4 carrying pLOW-*m(sf)gfp-cxaR*, ery[r]	This work
MDB445	NCTC8325-4 carrying pLOW-*clpX*, ery[r]	This work
MDB454	NCTC8325-4 carrying pLOW-*clpX-mCherry*, ery[r]	This work
MDB376	*cxaR-m(sf)gfp* (chromosomal), spc[r]	This work
MDB453	MDB376 carrying pLOW-*clpX-mCherry*, spc[r], ery[r]	This work
MDB326	NCTC8325-4, ∆*clpX*	[2]
EFS166	MDB326 carrying pLOW-*dCas9*, ery[r]	This work
EFS168	EFS166 carrying pVL2336-sgRNA(*cxaR*), ery[r], cam[r]	This work
EFS169	EFS166 carrying pVL2336-sgRNA(*luc*), ery[r], cam[r]	This work
MDB394	MDB326 carrying pLOW-*cxaR-m(sf)gfp*, ery[r]	This work
MDB435	MDB326 carrying pLOW-*cxaR*, ery[r]	This work
MDB446	MDB326 carrying pLOW-*clpX*, ery[r]	This work
MDB327	NCTC8325-4, $clpX_{I265E}$	[3]
EFS167	MDB327 carrying pLOW-*dCas9*, ery[r]	This work
EFS170	EFS167 carrying pVL2336-sgRNA(*cxaR*), ery[r], cam[r]	This work
EFS171	EFS167 carrying pVL2336-sgRNA(*luc*), ery[r], cam[r]	This work
MDB437	MDB327 carrying pLOW-*cxaR*, ery[r]	This work
MDB395	MDB327 carrying pLOW-*cxaR-m(sf)gfp*, ery[r]	This work
MK1465	NCTC8325-4 carrying pLOW-*dCas9*, ery[r]	Lab collection
EFS82	MK1465 carrying pVL2336-sgRNA(*clpX*), ery[r], cam[r]	This work
EFS84	MK1465 carrying pVL2336-sgRNA(*cxaR*), ery[r], cam[r]	This work
EFS68	MK1465 carrying pVL2336-sgRNA(*cxaR+clpX*), ery[r], cam[r]	This work
EFS88	MK1465 carrying pCG248-sgRNA(*luc*), ery[r], cam[r]	This work

EFS141	NCTC8325-4 carrying pAF256-*cxaR-SmBit/LgBit*, cam^r	This work
EFS145	NCTC8325-4 carrying pAP118-*cxaR-SmBit/clpP-LgBit*, cam^r	This work
EFS148	NCTC8325-4 carrying pAP118-*cxaR-SmBit/clpX-LgBit*, cam^r	This work
EFS177	NCTC8325-4 carrying pAP118-*cxaR-SmBit/cxaR-LgBit*, cam^r	This work
MDB384	NCTC8325-4 carrying pAP118-*cxaR-SmBit/sle1-LgBit*, cam^r	This work
MDB385	NCTC8325-4 carrying pAP118-*cxaR-SmBit/sle1$_{(no\ signal\ peptide)}$-LgBit*, cam^r	This work
MDB417	NCTC8325-4 carrying pAF256-*clpX-SmBit/LgBit*, cam^r	This work
MDB418	NCTC8325-4 carrying pAP118-*clpX-SmBit/sle1-LgBit*, cam^r	This work
MDB419	NCTC8325-4 carrying pAP118-*clpX-SmBit/sle1$_{(no\ signal\ peptide)}$-LgBit*, cam^r	This work
MDB420	NCTC8325-4 carrying pAP118-*clpX-SmBit/cxaR-LgBit*, cam^r	This work
MDB421	NCTC8325-4 carrying pAP118-*clpX-SmBit/clpP-LgBit*, cam^r	This work
MDB466	NCTC8325-4 carrying pAF256-*clpX$_{I265E}$-SmBit/LgBit*, cam^r	This work
MDB469	NCTC8325-4 carrying pAP118-*clpX$_{I265E}$-SmBit/cxaR-LgBit*, cam^r	This work
MDB470	NCTC8325-4 carrying pAP118-*clpX$_{I265E}$-SmBit/clpP-LgBit*, cam^r	This work
***S. aureus* JE2**		
JE2/MDB9	Community acquired MRSA strain, derivative of USA300 LAC cured of plasmids	[4]
MDB16	JE2 carrying pLOW-*dCas9_aad9*, spc^r	Lab collection
MHU44	MDB16 carrying pVL2336-sgRNA(*cxaR*), spc^r, cam^r	This work
MDB44	MDB16 carrying pCG248-sgRNA(*luc*), spc^r, cam^r	Lab collection
E. coli		
IM08B	DH10B, Δ*dcm*, P_{help}-*hsdMS*, P_{N25}-*hsdS* (strain expressing the *S. aureus* CC8 specific methylation genes)	[5]

a. cam^r = chloramphenicol resistant, spc^r = spectinomycin resistant, ery^r = erythromycin resistant.

References

1. Novick R. Properties of a cryptic high-frequency transducing phage in *Staphylococcus aureus*. Virology. 1967;33(1):155-66. doi: 10.1016/0042-6822(67)90105-5.
2. Frees D, Qazi SNA, Hill PJ, Ingmer H. Alternative roles of ClpX and ClpP in *Staphylococcus aureus* stress tolerance and virulence. Molecular Microbiology. 2003;48(6):1565-78. doi: 10.1046/j.1365-2958.2003.03524.x.
3. Stahlhut SG, Alqarzaee AA, Jensen C, Fisker NS, Pereira AR, Pinho MG, et al. The ClpXP protease is dispensable for degradation of unfolded proteins in *Staphylococcus aureus*. Scientific Reports. 2017;7(1):11739. doi: 10.1038/s41598-017-12122-y.
4. Fey Paul D, Endres Jennifer L, Yajjala Vijaya K, Widhelm Todd J, Boissy Robert J, Bose Jeffrey L, et al. A Genetic Resource for Rapid and Comprehensive Phenotype Screening of Nonessential *Staphylococcus aureus* Genes. mBio. 2013;4(1):10.1128/mbio.00537-12. doi: 10.1128/mbio.00537-12.
5. Monk IR, Tree JJ, Howden BP, Stinear TP, Foster TJ. Complete bypass of restriction systems for major *Staphylococcus aureus* lineages. mBio. 2015;6(3):e00308-15. Epub 20150526. doi: 10.1128/mBio.00308-15. PubMed PMID: 26015493; PubMed Central PMCID: PMCPMC4447248.
