# Supplementary material for: Cell splitting in Staphylococcus aureus is controlled by an adaptor protein facilitating degradation of a peptidoglycan hydrolase
Source: PLoS Genet. 2025 Sep 5;21(9):e1011841. doi: 10.1371/journal.pgen.1011841 (PMC12443321; doi:10.1371/journal.pgen.1011841)
Supplement: S4 Table — (PDF) [file pgen.1011841.s018.pdf]

S4 Table. Plasmids used in this work.

Name	Description[a]	Reference
pLOW	Low-copy number staphylococcal shuttle vector with a IPTG inducible P*spac* promoter and *lacI* repressor, amp^r, ery^r	[1]
pLOW-*dCas9_aad9*	For IPTG-inducible expression of dCas9, amp^r, spc^r	[2]
pLOW-*dCas9*_extra_*lacO*	For IPTG-inducible expression of dCas9, amp^r, ery^r	[3]
pLOW-*cxaR*	For IPTG-inducible expression of CxaR, amp^r, ery^r	This work
pLOW-*m(sf)gfp-cxaR*	For IPTG-inducible expression of CxaR with GFP fused to its N-terminus, amp^r, ery^r	This work
pLOW-*cxaR-m(sf)gfp*	For IPTG-inducible expression of CxaR with GFP fused to its C-terminus, amp^r, ery^r	This work
pLOW-*clpX*	For IPTG-inducible expression of CxaR, amp^r, ery^r	This work
pLOW-*clpX-mCherry*	For IPTG-inducible expression of CxaR with mCherry fused to its C-terminus, amp^r, ery^r	This work
pCG248	*E. coli*/*S. aureus* shuttle vector, amp^r, cam^r	[4]
pCG248-sgRNA(*luc*)	For constitutive expression of a nontargeting sgRNA, amp^r, cam^r	[3]
pVL2336	*E. coli*/*S. aureus* shuttle vector, amp^r, cam^r	[5]
pVL2336-sgRNA(*cxaR*)	For constitutive expression of sgRNA targeting *cxaR*, amp^r, cam^r	This work
pVL2336-sgRNA(*clpX*)	For constitutive expression of sgRNA targeting *clpX*, amp^r, cam^r	This work
pVL2336-sgRNA(*cxaR*+*clpX*)	For constitutive expression of sgRNA targeting both *cxaR* and *clpX*, amp^r, cam^r	This work
pVL2336-sgRNA(*SAOUHSC_00655*)	For constitutive expression of sgRNA targeting *SAOUHSC_00655*, amp^r, cam^r	This work
pVL2336-sgRNA(*SAOUHSC_00656*)	For constitutive expression of sgRNA targeting *SAOUHSC_00656*, amp^r, cam^r	This work
pVL2336-sgRNA(*SAOUHSC_00658*)	For constitutive expression of sgRNA targeting *SAOUHSC_00658*, amp^r, cam^r	This work
pVL2336-sgRNA(*SAOUHSC_00660*)	For constitutive expression of sgRNA targeting *SAOUHSC_00660*, amp^r, cam^r	This work
pVL2336-sgRNA(*S245*)	For constitutive expression of sgRNA targeting *S245*, amp^r, cam^r	This work
pVL2336-sgRNA(*sle1*)	For constitutive expression of sgRNA targeting *sle1*, amp^r, cam^r	This work
pMAD	Thermosensitive shuttle vector for allelic replacement in Gram-positive bacteria, amp^r, ery^r	[6]
pMAD-*cxaR::spc*	To knock out *cxaR* by allelic replacement, amp^r, ery^r	This work
pMAD-*cxaR-m(sf)gfp_spc*	To chromosomally integrate GFP-tagged *cxaR* in its native locus, amp^r, ery^r	This work
pAF256-P_{tet}-*hupA-smbit/lgbit*	Split-luciferase control plasmid, originally for assessing HupA self-interaction in *C. difficile*, cam^r	[7]
pAF256-*cxaR-smbit/lgbit*	CxaR split-luciferase control, cam^r	This work
pAF256-*clpX-smbit/lgbit*	ClpX split-luciferase control, cam^r	This work
pAF256-$clpX_{I265E}$-*smbit/lgbit*	$ClpX_{I265E}$ split-luciferase control, cam^r	This work
pAP118-P_{tet}-*hupA-smbit/hupA-lgbit*	Split-luciferase test plasmid, originally for assessing HupA self-interaction in *C. difficile*, cam^r	[7]
pAP118-*cxaR-smbit/clpP-lgbit*	For assessing interactions between CxaR and ClpP, cam^r	This work
pAP118-*cxaR-smbit/clpX-lgbit*	For assessing interactions between CxaR and ClpX, cam^r	This work
pAP118-*cxaR-smbit/cxaR-lgbit*	For assessing CxaR self-interaction, cam^r	This work

pAP118-*cxaR-smbit/sle1-lgbit*	For assessing interactions between CxaR and Sle1, cam^r	This work
pAP118-*cxaR-smbit/sle1*$_{(no\ signal\ peptide)}$-*lgbit*	For assessing interactions between CxaR and Sle1, cam^r	This work
pAP118-*clpX-smbit/sle1-lgbit*	For assessing interactions between ClpX and Sle1, cam^r	This work
pAP118-*clpX-smbit/sle1*$_{(no\ signal\ peptide)}$-*lgbit*	For assessing interactions between ClpX and Sle1, cam^r	This work
pAP118-*clpX-smbit/cxaR-lgbit*	For assessing interactions between ClpX and CxaR, cam^r	This work
pAP118-*clpX-smbit/clpP-lgbit*	For assessing interactions between ClpX and ClpP, cam^r	This work
pAP118-*clpX*$_{I265E}$-*smbit/cxaR-lgbit*	For assessing interactions between $ClpX_{I265E}$ and CxaR, cam^r	This work
pAP118-*clpX*$_{I265E}$-*smbit/clpP-lgbit*	For assessing interactions between $ClpX_{I265E}$ and ClpP, cam^r	This work

a. amp^r = ampicillin resistant, ery^r = erythromycin resistant, spc^r = spectinomycin resistant, cam^r = chloramphenicol resistant.

References

1. Liew ATF, Theis T, Jensen SO, Garcia-Lara J, Foster SJ, Firth N, et al. A simple plasmid-based system that allows rapid generation of tightly controlled gene expression in *Staphylococcus aureus*. Microbiology (Reading, England). 2011;157(3):666-76. doi: 10.1099/mic.0.045146-0.
2. Myrbråten IS, Stamsås GA, Chan H, Angeles DM, Knutsen TM, Salehian Z, et al. SmdA is a Novel Cell Morphology Determinant in *Staphylococcus aureus*. mBio. 2022;13(2):e03404-21. doi: 10.1128/mbio.03404-21.
3. Stamsås GA, Myrbråten I, Straume D, Salehian Z, Veening J-W, Håvarstein LS, et al. CozEa and CozEb play overlapping and essential roles in controlling cell division in *Staphylococcus aureus*. Mol Microbiol. 2018;109(5):615-32. doi: 10.1101/256560.
4. Helle L, Kull M, Mayer S, Marincola G, Zelder M-E, Goerke C, et al. Vectors for improved Tet repressor-dependent gradual gene induction or silencing in *Staphylococcus aureus*. Microbiology (Reading, England). 2011;157(12):3314-23. doi: 10.1099/mic.0.052548-0.
5. Liu X, Bakker Vd, Heggenhougen MV, Mårli MT, Frøynes AH, Salehian Z, et al. Genome-wide CRISPRi screens reveal the essentialome and determinants for susceptibility to dalbavancin in *Staphylococcus aureus*. bioRxiv. 2023:2023.08.30.555613. doi: 10.1101/2023.08.30.555613.
6. Arnaud M, Chastanet A, Debarbouille M. New vector for efficient allelic replacement in naturally nontransformable, low-GC-content, Gram-positive bacteria. Appl Environ Microbiol. 2004;70(11):6887-91. doi: 10.1128/AEM.70.11.6887-6891.2004. PubMed PMID: 15528558; PubMed Central PMCID: PMCPMC525206.
7. Oliveira Paiva AM, Friggen AH, Qin L, Douwes R, Dame RT, Smits WK. The Bacterial Chromatin Protein HupA Can Remodel DNA and Associates with the Nucleoid in *Clostridium difficile*. Journal of Molecular Biology. 2019;431(4):653-72. doi: 10.1016/j.jmb.2019.01.001.
