# Supplementary material for: Cell splitting in Staphylococcus aureus is controlled by an adaptor protein facilitating degradation of a peptidoglycan hydrolase
Source: PLoS Genet. 2025 Sep 5;21(9):e1011841. doi: 10.1371/journal.pgen.1011841 (PMC12443321; doi:10.1371/journal.pgen.1011841)
Supplement: S5 Table — (PDF) [file pgen.1011841.s019.pdf]

S5 Table. Oligonucleotide primers used in this work.

Name	Sequence 5'-3'[a]	Description[b]
**Primers for construction of pMAD-*cxaR::spc***		
mk188	ATTGGGCCCACCTAGGATC	*aad9*_up F
mk189	ACTATGCGGCCGCTCGAG	*aad9*_down R
mk591	ATCGCCATGGGCAGATAATACAAAAGATATGGT	*cxaR*_up F w/NcoI
mk592	**GATCCTAGGTGGGCCCAAT**CGTACTAAATCAAACTCGCCA	*cxaR*_up R
mk593	**CTCGAGCGGCCGCATAGT**ATTACACCAACGTAATGCACG	*cxaR*_down F
mk594	ATCGGGATCCTCGCTTCAAAGGCAGTACCA	*cxaR*_down R w/BamHI
**Primers for construction of pMAD-*cxaR-m(sf)gfp_spc***		
mdb58	ACCTCCATGGGATCCAGGTGCTCAAAGTATG	*cxaR*_up F w/NcoI
mdb59	ATGTTTTTTTATAAGGTGTTGCAT	*cxaR*_up R
mdb60	ATGCAACACCTTATAAAAAAACAT	*cxaR-m(sf)gfp* F
mdb61	**TTTCCGTTAATCAAATTGCTCAT**TTACTTATAAAGCTCATCCATGC	*cxaR-m(sf)gfp* R
mdb62	**CTATAGAAACTTCTCTCAATTAG**TTACATTTCAATTATATTAGCTTAAT	*cxaR*_down F
mdb63	AGGTGTCGACCCAAACCAGATTCCCTTCCA	*cxaR*_down R w/SalI
mk503	ATGAGCAATTTGATTAACGGAAA	*spc* F
mk504	CTAATTGAGAGAAGTTTCTATAG	*spc* R
**Primers for screening of pMAD plasmids**		
im156	AATCTAGCTAATGTTACGTTACA	pMAD F
mk177	GATGCCGCCGGAAGCGAG	pMAD R
**Primers for creating pLOW constructs**		
mhu26	ACGTGGATCCCAACACCTTATAAAAAAACATGTA	*cxaR* F w/BamHI
mhu27	ACGTGAATTCATGTAATTAACGTGCATTACGTTG	*cxaR* R w/EcoRI
mhu28	ATCCTCGAGTCGACCAATAAAACT*AGGAGG*AAATTTAAATGCAACACCTTATAAAAAAACAT	*cxaR* F w/XhoI+SalI
mhu29	TCCGGGGATCCGACGTCCATTACGTTGGTGTA	*cxaR* R w/BamHI
mdb98	ATCCTCGAGGTCGACCAATAAAACT*AGGAGG*AAATTTAAATGTTTAAATTCAATGAAGATGAA	*clpX* F w/XhoI+SalI
mdb99	ACGTGAATTCCAATGATTAAGCTGATGTTTTAC	*clpX* R w/EcoRI
mdb102	TCCGGGGATCCAAGCTGATGTTTTACTATTATTAATT	*clpX* R w/BamHI
**Primers for screening of pLOW plasmids**		
im218	TCTCATTCAATTCCTAGGTGG	pLOW F
im134	TGTGCTGCAAGGCGATTAAG	pLOW R
**Oligos for creating pVL2336-sgRNA constructs**		
SAOUHSC_00427_for	TATAATTGCCCACACTGATTCACC	*sle1* sgRNA F
SAOUHSC_00427_rev	AAACGGTGAATCAGTGTGGGCAAT	*sle1* sgRNA R
SAOUHSC_00655_for	TATATAGCAATCAGATCTAACTCT	_*00655* sgRNA F
SAOUHSC_00655_rev	AAACAGAGTTAGATCTGATTGCTA	_*00655* sgRNA R
SAOUHSC_00656_for	TATACACGAACCATGTTAACCCCG	_*00656* sgRNA F
SAOUHSC_00656_rev	AAACCGGGGTTAACATGGTTCGTG	_*00656* sgRNA R
SAOUHSC_00658_for	TATACACTTGCAATTTCTTTACTG	_*00658* sgRNA F
SAOUHSC_00658_rev	AAACCAGTAAAGAAATTGCAAGTG	_*00658* sgRNA R
SAOUHSC_00659_for	TATAAAATTCCATAAAATCTGTTT	*cxaR* sgRNA F
SAOUHSC_00659_rev	AAACAAACAGATTTTATGGAATTT	*cxaR* sgRNA R

SAOUHSC_ S245_for	TATATTTTAAGTATTAAAAAAGGC	*S245* sgRNA F
SAOUHSC_ S245_rev	AAACGCCTTTTTTAATACTTAAAA	*S245* sgRNA R
SAOUHSC_ 00660_for	TATAGCCCACTACAATGCCGACGT	_*00660* sgRNA F
SAOUHSC_ 00660_rev	AAACACGTCGGCATTGTAGTGGGC	_*00660* sgRNA R
SAOUHSC_ 01778_for	TATATCCATAATTTCTTTAGGAGT	*clpX* sgRNA F
SAOUHSC_ 01778_rev	AAACACTCCTAAAGAAATTATGGA	*clpX* sgRNA R
**Primers for screening of pCG248 and pVL2336 plasmids**		
mk26	GGATAACCGTATTACCGCCT	pCG248 F
mk25	AAATCTCGAAAATAATAGAGGGA	pCG248 R
**Primers for creating split-luciferase constructs**		
efs7	TCAGAGCTCATCTGGAGAAAT*AGGAGG*AC	*cxaR* F w/SacI
efs8	GCCCTCGAGAACGTGCATTACGTTGGTGTA	*cxaR* R w/XhoI
mdb93	ATCGGAGCTCCAATCTAGTATAGTCTTTAACG	*clpX* F w/SacI
mdb94	GCCCTCGAGCAGCTGATGTTTTACTATTATTAAT	*clpX* R w/XhoI
efs5	GTCCGATCGCAATCTAGTATAGTCTTTAACG	*clpX* F w/PvuI
efs6	GGCGGCCGCAGCTGATGTTTTACTATTATTAAT	*clpX* R w/NotI
efs9	GTCCGATCGGTAACAGTTATTACA*AGGAGG*	*clpP* F w/PvuI
efs10	GGCGGCCGCTTTTGTTTCAGGTACCATCAC	*clpP* R w/NotI
efs20	GTCCGATCGATCTGGAGAAAT*AGGAGG*AC	*cxaR* F w/PvuI
efs21	GGCGGCCGCACGTGCATTACGTTGGTGTA	*cxaR* R w/NotI
mdb88	ATCGCGATCGGTTAAGCAAG*AGGAGG*ATTTTA	*sle1* F w/PvuI
mdb89	GCCGCGGCCGCGTGAATATATCTATAATTATTTACTT	*sle1* R w/NotI
mdb90	ATCGCGATCGCAATAAAACTA*GGAGG*AAATTTAAATGGCTACAACTC ACACAGTAAAAC	*sle1* F w/PvuI
**Primers for screening of split-luciferase constructs**		
efs11	CATGCCAATACAATGTAGGC	Split-luciferase F
efs53	AGACTTCTCATGAGAGAAGC	Split-luciferase R

a. The restriction sites are underlined, the overhangs are bolded, and the ribosomal binding sites are italicized.
b. F = forward primer, R = reverse primer, RS = restriction site, and RBS = ribosomal binding site.
